# Are Forensic Experts Already Biased before Adversarial Legal Parties Hire Them?

**DOI:** 10.1371/journal.pone.0154434

**Published:** 2016-04-28

**Authors:** Tess M. S. Neal

**Affiliations:** School of Social & Behavioral Sciences, New College of Interdisciplinary Arts & Sciences, Arizona State University, Phoenix, Arizona, United States of America; TNO, NETHERLANDS

## Abstract

This survey of 206 forensic psychologists tested the “filtering” effects of preexisting expert attitudes in adversarial proceedings. Results confirmed the hypothesis that evaluator attitudes toward capital punishment influence willingness to accept capital case referrals from particular adversarial parties. Stronger death penalty opposition was associated with higher willingness to conduct evaluations for the defense and higher likelihood of rejecting referrals from all sources. Conversely, stronger support was associated with higher willingness to be involved in capital cases generally, regardless of referral source. The findings raise the specter of skewed evaluator involvement in capital evaluations, where evaluators willing to do capital casework may have stronger capital punishment support than evaluators who opt out, and evaluators with strong opposition may work selectively for the defense. The results may provide a partial explanation for the “allegiance effect” in adversarial legal settings such that preexisting attitudes may contribute to partisan participation through a self-selection process.

## Introduction

Adversarial legal systems, such as the American system, are designed so that two advocates represent their parties’ respective positions before an impartial trier-of-fact (typically a judge or jury), whose task is to ferret out truth and make a binding decision to provide justice [1]. Rule 702 of the Federal Rules of Evidence [2] allows expert witnesses to testify in a case if they have scientific, technical, or other specialized knowledge that would help the trier-of-fact better understand a particular piece of evidence or reach a determination about a contested fact, so long as the expert’s methods and application to the particular case were appropriate. The law, and the various professions in which these experts are trained, generally presume that these experts will be impartial, participating as an objective expert by interpreting data on its own strength, rather than in a biased manner reflecting which side hired them.

However, a growing body of research demonstrates the biasing effects of the adversarial legal system on experts. Most of these studies are field studies that show evidence of forensic expert partiality in patterns of data from actual cases, but they cannot explain the reason for that partiality because they are not true experiments (e.g., [3–7]). The experts’ partiality might be explained in many ways. For instance, the socialization of the expert witness to the case could be affected by the retaining party (i.e., the adversarial process corrupts a neutral expert). Or perhaps the bias may exist in the attitudes of forensic experts before referrals are even accepted; that is, the expert may be partial and self-select or filter adversarial involvement before the adversarial process starts. Or attorneys might choose to retain evaluators who have preexisting attitudes that favor their side, or call only those experts with favorable findings to testify in court [8].

One research team recently conducted an experimental study to isolate the causal link between adversarial side and a forensic experts’ biased interpretation of data. Murrie, Boccaccini, Guarnera, and Rufino [9] had forensic mental health professionals conduct a “file review” of a case, leading the experts to believe this was an actual case rather than a research study. The sole experimental manipulation was referral source (defense or prosecuting attorney), and all the other information was held constant. The forensic experts were asked to review information about the offender and score two common and well-researched risk assessment tools. The results provided strong evidence of “adversarial allegiance,” or the tendency for experts to reach conclusions that support the retaining party’s position. Specifically, experts who thought they were working for the prosecution assigned higher risk scores to offenders and experts who thought they were working for the defense assigned lower risk scores to those same offenders, with effect sizes (*d*) ranging up to 0.85. These results indicate that the first explanation in the preceding paragraph was correct–that presumably neutral experts were “corrupted” simply by knowing from whom the referral came.

But is that the only possible explanation for forensic expert partiality? Perhaps there are multiple, non-mutually-exclusive factors underlying apparent expert partiality. Basic research in psychological science tells us that elements of the situation interact with elements about a given person to predict behavior [10], and people choose to enter and spend time in situations that reflect features of their personalities and promote the expression of their social attitudes [11–12]. The Murrie et al. study [9] provides compelling information about the power of the situation–the adversarial context itself–on expert interpretation of data. But what about the unique characteristics of the experts themselves? In addition to the demonstrated influence of the adversarial system on forensic expert partiality, might attorney and expert selection effects also occur due to the unique characteristics of the experts, with the potential to amplify expert partiality in the adversarial system? For instance, biased experts retained by an adversarial party may then be influenced by the retaining attorney’s theory of the case, further skewing their perspective.

The present study addresses one of these questions. Specifically, it examines *evaluator self-selection* as a complementary explanation for expert partiality in adversarial legal settings via a “filtering” process. To answer this question, we measured the death penalty attitudes of 206 forensic psychologists and asked them whether they would be willing to accept referrals in capital cases from the defense, prosecution, and court. If death penalty attitudes are shown to be systematically related to willingness to accept capital cases from particular referrals sources, it will demonstrate that preexisting expert bias may contribute to expert partiality in the adversarial legal system through a self-selection “filtering” process of involvement in cases.

Importantly, any evidence of “filtering” of forensic mental health experts in capital cases is but one particular context in which this type of “filtering” phenomenon may occur. We use this one context as an initial example, but assert that if evidence of filtering or self-selecting effects are found in this context that these filtering effects can likely be found in other contexts as well, with other kinds of forensic experts. This self-selection effect is not presumed to be specific to mental health experts or to capital cases. For instance, product experts in medical malpractices cases might make decisions about whether to take a case from the plaintiff or defense based on their preexisting beliefs about tort law generally. Experts in these cases might selectively work for insurance firms on defense if they believe tort law has grown out of control and support the tort reform movement, and other experts might selectively choose to work for the plaintiff if they believe tort litigation continues to play an important role in our society.

### Forensic Mental Health Evaluations in Capital Cases

Capital punishment is one of the most fiercely debated issues in American society. It is a powerful legal, ethical, and moral issue about which many people have strongly held opinions. In capital cases, mental health practitioners may be asked to evaluate a defendant’s mental health to help the court adjudicate the case. Thus, it is possible that in capital case evaluations, examiners’ attitudes toward capital punishment might influence their willingness to become involved or the specific ways in which they would become involved in the adversarial process.

A variety of different forensic evaluations can be considered “capital case” referrals, from insanity pleas, to capital mitigation, capital sentencing risk assessments, competence for execution evaluations, and evaluations of intellectual disability. For example, for a possible insanity plea, a clinician could be asked to assess whether the defendant exhibited symptoms of mental illness at the time of the alleged crime to help the court determine whether the defendant should be found Not Guilty by Reason of Insanity.

In capital mitigation cases, the assessor could report on factors that could reduce the severity of punishment from death down to life in prison, such as a defendant’s history of abuse or lack of prior violence or criminal record. In capital sentencing risk assessments, clinicians are often asked to help the court decide whether the evaluee poses a future danger to society. For inmates sentenced to death, clinicians might be asked long after the sentencing date whether the inmate is competent to move forward with a pending execution. And for Atkins-type evaluations, clinicians are asked to help the court determine whether a capital defendant is intellectually disabled (formerly called mental retardation), because the U.S. Supreme Court decided in 2002 that executing an intellectually disabled individual would violate the Eighth Amendment’s ban on cruel and unusual punishment [13].

#### Evaluator attitudes toward capital punishment and involvement in capital cases

The strength of an evaluator’s opinions toward the death penalty may influence whether and how clinicians become involved in capital case work. For instance, evaluators who strongly oppose the death penalty report being significantly less likely to accept a Competency for Execution (CFE) referral [14–15]. These findings suggest there are self-selection factors in some capital case evaluations, in which the evaluators who take CFE referrals may be more supportive of the death penalty than evaluators who decline.

Haney [16] argued that “death qualification” of juries facilitates death sentencing because only people who support capital punishment are allowed to have a say in deciding whether any capital defendant lives or dies. This is because the United States Supreme Court decided that in order to serve in a capital case, jurors must be willing to consider all sentencing options; that is, if their opinions would prevent them from considering death, they are not “death qualified” and must be stricken from the jury [17–18]. Haney points out, “capital juries can only represent the conscience of one part of the community–the part that collectively tilts toward death” (p. 139). If a similar process occurs in experts, and self-selection leads to death-qualified psychologists being the only (or primary) professionals who are evaluators in some kinds of capital cases, the pool of potential examiners may misrepresent the range of examiner attitudes toward capital punishment. In addition to concern about the strength of an examiner’s personal support for the death penalty, Cunningham and Reidy [19] argue that the stronger one’s opposition, the more concerns about objectivity are increased. They suggest that mental health professionals who are strong advocates against capital punishment may find themselves in an ethically compromised dual-role if they are hired as experts in capital cases.

### The Current Project

The research to date has surveyed forensic clinicians about whether they would accept a referral in a particular kind of case, without assessing from whom the clinician would consider accepting the referral (such as CFE evaluations [14–15]). However, the referral party theoretically matters in capital cases. The “direction” of capital punishment attitude bias can be predicted to fall along adversarial party lines, such that clinicians who more strongly support the death penalty may be more likely to accept capital case referrals from the prosecution whereas clinicians who strongly oppose the death penalty may be more likely to accept defense referrals. This hypothesis–that evaluators who more strongly support the death penalty will be more likely to accept capital case referrals from the prosecution, whereas those who oppose the death penalty will be more likely to accept capital case referrals from the defense–is the central hypothesis of the current study.

Of note, this hypothesis is invariant to the “type” of capital case referral. That is, evaluators who strongly support capital punishment are hypothesized to be more likely to accept a capital case referral from the prosecution in insanity evaluations, in risk assessments, in CFE evaluations, and in Atkins-type evaluations. The reverse is hypothesized for evaluators who hold strong anti-capital punishment attitudes. They would theoretically be more likely to accept a capital case referral from the defense in insanity evaluations, mitigation evaluations, risk assessments, CFE evaluations, and Atkins-type evaluations.

This study also tests the supplemental *a priori* hypothesis that examiners willing to accept capital case referrals (i.e., get involved in capital cases generally) will have stronger death penalty support than those who abstain. This hypothesis parallels for expert witnesses Haney’s [16] “death qualification” hypothesis about jurors in capital cases. Our aims for this study were achieved; we were able to test both the central and supplemental hypotheses, as detailed below.

## Method

### Participants

The participants in this study consisted of practicing forensic psychologists in the U.S.A. The American Psychological Association (APA) website directory was utilized in an attempt to generate 1000 randomly selected participants who were clinical-forensic psychologists. It was unclear how many clinical-forensic psychologists were available in the population from which to select, and only 962 psychologists with clinical-forensic interests could be identified through the APA directory. Thus, surveys were mailed to this population of 962 psychologists. Previous research with both APA members and those who are not APA members indicate APA membership is sufficiently representative of doctoral-level clinicians with respect to demographic characteristics, education, and employment to use the member database for research purposes [20].

Of the 962 surveys mailed, 351 were completed. Other large national surveys of clinical-forensic psychologists in the U.S.A. have yielded similar sample sizes (e.g., *N* = 434 in Neal & Grisso [21]). Of the 351 completed surveys, 249 of the respondents reported practicing in a jurisdiction with the death penalty. Of these 249 participants, only 206 provided complete answers to the “willingness to accept referral” questions. Thus, for the purposes of this paper, analyses are restricted to the 206 participants who practice in death penalty jurisdictions and who answered the critical dependent variables. Respondents included forensic psychologists from all 31 states with the death penalty, plus 4 additional states that still had the death penalty as of the time these data were collected in late 2010 (CT, IL, MD, and NE) [22], as well as psychologists practicing in the federal system.

Most participants were White (91.6%; 4.8% Hispanic, 0.8% Asian-American, 0.4% African-American, 2.4% Other). The average age was 58.84 (*SD* = 9.42) and approximately three-quarters were men (72%). The majority had a Ph.D. (86%; 13% Psy.D.; 1% Other). Participants reported an average of 22.24 years of conducting forensic evaluations (*SD* = 9.39). Approximately 28% reported being board-certified. Most participants reported working in private practice or for private agencies (60%), followed by public sector institutions or agencies (17%), and university settings (10%; 13% “other or more than one”).

### Procedure

Approval was sought from and provided by the University of Alabama Institutional Review Board for the Protection of Human Subjects prior to beginning this work. The survey packets were mailed because surveys conducted via postal mail produce higher response rates than electronic mail [23]. The mailed packet included a cover letter indicating the research was being conducted by a university graduate student, an Institutional Review Board informed consent sheet, the set of questionnaires printed on green paper, and a separate debriefing page. Also enclosed were a self-addressed stamped envelope with first-class outgoing postage and a one-dollar bill as gesture of appreciation for participation. A follow-up postcard was sent two weeks after the initial mailings to express appreciation to those who had responded and to remind those who had not responded about the survey. Prior studies have demonstrated that each of these methods have independent effects on increasing response rates in postal surveys [24–25].

### Measures

#### Forensic psychologist questionnaire

Developed for use in this study, this questionnaire included questions about highest degree earned, specialty board certification, primary place of employment, number of years conducting forensic evaluations, jurisdictions of practice, and death penalty status in those jurisdictions. For those evaluators practicing in jurisdictions with the death penalty, they were asked, “Have you or would you evaluate a defendant in a capital case for the prosecution?” as well as “…for the defense?” and “…for the court as a court-appointed assessor?” The questionnaire also included demographic race, age, and gender items.

#### Death Penalty Attitudes Scale (DPAS)

O’Neil, Patry, and Penrod [26] developed the 15-item DPAS to measure attitudes toward the death penalty. Items are answered on a nine-point Likert-type scale (1, *strongly disagree*, to 9, *strongly agree*) with higher scores indicating greater death penalty support. Although the scale was initially designed to measure jurors’ death penalty attitudes, the scale has been found to correlate highly (*r* > 0.85) with other measures of death penalty support and has demonstrated strong psychometric properties in previous research. Results were used to obtain quantitative data regarding the relative strength of the forensic psychologists’ attitudes toward the death penalty. The DPAS evidenced good reliability in this sample; α = 0.84, average inter-item correlation = 0.27. As far as we are aware, this is the first study to use the DPAS to measure forensic psychologists’ death penalty attitudes (and may be the first study to ever measure forensic psychologists’ death penalty attitudes).

The average DPAS score among forensic psychologists for all valid responses in our overall sample (*n* = 329) was 3.16 (*SD* = 1.25, range 1–7). The average DPAS score among the 206 cases analyzed in this paper was 3.08 (*SD* = 1.22, range = 1–6.33). These values are lower than the values reported by O’Neil et al. [26] in the original validation article of the DPAS across 11 studies. With a total combined sample size of 2,849 people across those 11 studies, O’Neil et al. provided data about DPAS scores with a mean value of 4.83 –a higher value than in the forensic psychologists who responded to our survey. However, there are two subsamples of the O’Neil et al. data that are more relevant–and closer in mean value–to our participants. The mean DPAS score for a subsample of the highly educated people their overall large sample (“post-college education”) was 4.14. And in a subsample of 127 older adults (“age 50+”) from O’Neil et al.’s overall large sample, the mean DPAS value was 4.32.

## Results

Multinomial logistic regression was used to analyze the central hypothesis because the predictor variable was continuous (death penalty attitude score) and the dependent variable was a nominal variable with five mutually-exclusive outcome groups. Each participant was only in one outcome group, depending on from which source(s) they reported they would accept capital referrals. The five outcome groups included forensic psychologists who would accept referrals in capital cases from the defense or defense and court only (i.e., reject only the prosecution); from the prosecution or prosecution and court only (i.e., reject only the defense); from only the court (i.e., reject only the adversarial referrals); accept from all of the three sources; or accept from no sources at all.

Although we wanted to analyze the data by “defense only,” “prosecution only,” “court only,” “all,” and “none,” there were not enough participants who fell into the “defense only” (*n* = 3) and “prosecution only” (*n* = 0) groups to move forward with the analysis. Thus, we combined participants who would accept only from the defense with those who would accept from the defense and the court (but would reject the prosecution), calling this the “accept from defense or defense-and-court only” group, which added 15 more participants for a total of 18 in this group. We did the same for prosecution only + prosecution-and-court-only, but there were no participants in either subgroup. Descriptive statistics are provided in Table 1.

**Table 1:** Descriptive Statistics.

Outcome Group ^a^	*N*	DPAS		
		*M*	*SD*	Range
Accept Defense or Defense-and-Court Only	18	2.04	0.99	1.00–5.27
Accept Prosecution or Prosecution-and-Court Only	0	—	—	—
Accept Court Only	6	3.24	0.75	1.80–3.93
Accept From All Three Sources	140	3.33	1.17	1.00–6.33
Accept None (Refuse All Three Sources)	42	2.69	1.23	1.00–5.73

^a^ Outcome group is a mutually exclusive category–each participant is only in one group, depending on from which source(s) they reported they would accept capital referrals. DPAS = score on the Death Penalty Attitudes Scale, with higher scores indicating greater support for the death penalty (range 1–9).

**Fig 1:**
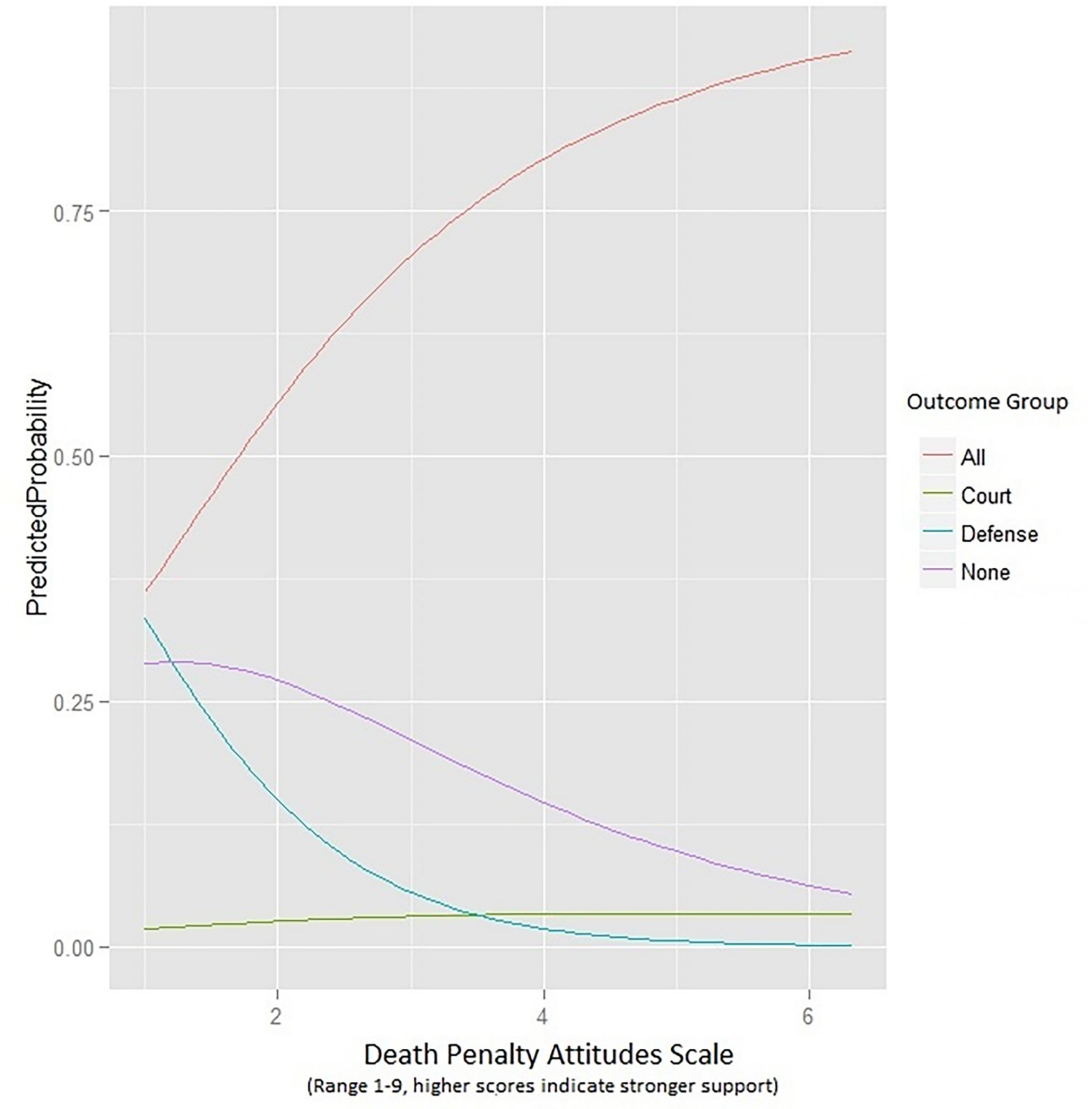
Multinomial Logistic Regression Graph of Death Penalty Support and Predicted Probability of Referral Acceptance from Mutually-Exclusive Groups. The reference category is Willingness to Accept from Defense or Defense-and-Court Only (i.e., Reject only the Prosecution), which is labeled “Defense” here. “All” = willing to accept from all three referral sources, “Court” = willing to accept only from the court, and “None” = not willing to accept from any of the three referral sources. No participants reported they would accept from the prosecution only, and thus they are not represented on this graph. DPAS = score on the Death Penalty Attitudes Scale, with higher scores indicating greater support for the death penalty (range 1–9). The sample sizes for the red “all” and purple “none” lines are higher and thus are likely more stable representations of the underlying phenomena than the green “court” and blue “defense” lines that represent fewer points of data.

Results are provided in Table 2 and Fig 1, indicating that the central hypothesis was partially supported. The first part of the hypothesis–that forensic evaluators with stronger death penalty support would be more likely to accept capital case referrals solely from the prosecution–was not supported. This null result occurred because not a single forensic psychologist reported that they would only work for the prosecution (or even for just the prosecution and court). In contrast, 9% of the sample reported they would work only for the defense (or defense-and-court only), 2% would work only for the court, 20% would refuse all capital referrals regardless of source, and 68% would accept the referrals from any of the three sources.

**Table 2:** Death Penalty Support Multinomial Logistic Regression Results.

Outcome Group	*N*	*Β*	*SE*	*p*	OR	95% CI
Accept Defense or Defense-and-Court Only	18	*(reference category)*				
Accept Prosecution or Prosecution-and-Court Only	0	—	—	—	—	—
Accept Court Only	6					
Intercept		-4.12	1.38	.003	—	—
DPAS		1.18	.47	.011	3.25	1.31–8.10
Accept All	140					
Intercept		-1.16	.75	.122	—	—
DPAS		1.24	.33	< .001	3.45	1.82–6.52
Accept None (Refuse All)	42					
Intercept		-.90	.80	>.250	—	—
DPAS		.75	.34	.028	2.11	1.09–4.11

The reference category is Willingness to Accept from Defense or Defense-and-Court Only (i.e., Reject only the Prosecution). OR is the odds ratio. DPAS = score on the Death Penalty Attitudes Scale, with higher scores indicating greater support for the death penalty (range 1–9). The predictor variable is score on the Death Penalty Attitudes Scale. The dependent variable is one of five mutually exclusive outcome groups.

The second part of the central hypothesis was supported. As predicted, lower death penalty support was associated with a higher probability of accepting defense/defense-and-court-only referrals compared to accepting referrals from the court only (odds ratio = 3.25; 95% CI 1.31–8.10), from any of the three sources (3.45; 95% CI 1.82–6.52), and from none of the three sources (2.11; 95% CI 1.09–4.11). These results mean that each one-unit increase in death penalty support as measured by the DPAS more than tripled the odds of an examiner accepting capital referrals solely from the court, more than tripled the odds of accepting from any source, and more than doubled the odds of refusing all capital referrals, compared to accepting from defense or defense-and-court only (i.e., rejecting only the prosecution).

Fig 1 graphically depicts these results. The red accept “all” referrals line at the top of the graph shows that increasing death penalty support (on the X axis) is associated with a higher predicted probability of belonging to the group of evaluators who would agree to accept capital referrals from any source. The purple “none” line in the middle of the graph shows that lower death penalty support is associated with a higher predicted probability of belonging to the group of evaluators who would not accept any capital referrals. The blue “defense” line is similar to the purple “none” line in that lower death penalty support is associated with a higher predicted probability of belonging to the group of evaluators who would accept referrals only from the defense or defense-and-court. Finally, the flat green “court” line at the bottom of the graph shows that death penalty attitudes are not associated with likelihood of belonging to a group of evaluators who would only work for the court in capital cases. Although the “court” line is flat, the multinomial logistic regression result is significant because it compares the slope of that line to the slope of the “defense” line, finding a difference in the two slopes relative to one another (see Table 2 and paragraph above). It should be noted that more confidence can be placed in the slope of the red “all” and purple “none” lines than the green “court” and blue “defense” lines, given the subsample sizes in this dataset.

The supplemental “death qualification” hypothesis was supported. Examiners willing to accept capital case referrals from any source (i.e., get involved in capital cases generally) had significantly higher DPAS scores than examiners who would refuse to be involved in a capital case in any capacity, *t* (204) = 2.40, *p* = 0.017, Cohen’s *d* = 0.34 (*M* = 3.19, *SD* = 1.20, *n* = 164 and *M* = 2.69, *SD* = 1.23, *n* = 42, respectively). This effect is amplified by comparing the evaluators who would explicitly work for any capital case referral source–those who would “opt in” without the adversarial filtering effect (*M* = 3.33, *SD* = 1.17, *n* = 140)–compared to those would fully abstain, *t* (180) = 3.07, *p* = 0.003, Cohen’s *d* = 0.46.

## Discussion

These are the first data to test the “filtering” effects of expert self-selection in adversarial cases by documenting the association between expert attitudes and willingness to accept a referral from a particular adversarial source. Filtering and selection effects in adversarial settings have been assumed to exist, but with few empirical tests of the hypothesis to date. The present results provide some evidence that filtering at various stages of the forensic referral system can introduce systematic bias into the adversarial legal system. Evaluator attitudes and other attributes may systematically influence from whom evaluators are willing to accept a referral in legal cases.

The present study complements the Murrie and colleagues [9] experimental study that showed that the adversarial system induces bias in presumably neutral experts. The current study demonstrated that these experts have *preexisting* biases that may affect for whom they are willing to work in the adversarial system–thus, likely amplifying the effects of the system-induced biases when layered with preexisting expert biases. Together, the Murrie et al. findings and the findings of the present investigation can be situated in basic psychological science. Consistent with Lewin’s [10] theory that unique characteristics of persons interact with the unique characteristics of situations to produce behavior, the data from these studies demonstrate that both the adversarial system itself (i.e., the situation; Murrie et al) and unique characteristics of the person (death penalty attitudes in this case, demonstrated in the present study) affect expert behavior.

Evaluators who do not support capital punishment are more likely to prefer defense referrals, and those with higher support for capital punishment are more likely to be involved in capital cases generally. We expected that experts with strong support for the death penalty would be more likely to accept prosecution-only referrals, but there were no experts who would accept capital referrals from only the prosecution this sample of 206 forensic experts. And the mean DPAS score–even among the group of evaluators who “more strongly support” capital punishment is still low on that scale–indicating that forensic psychologists as a group appear to have low support for the death penalty. Although the data show those who agree to participate have stronger support for the death penalty than those who decline to be involved, “stronger support” essentially boils down to “smaller dislike” than support *per se* (though the range shows there is some variability in the data). Importantly, however–and relevant to the “filtering effect” question–there is a subset of forensic experts with very low support for capital punishment who choose to be involved selectively in defense work in capital cases, revealing the likelihood of preexisting bias affecting adversarial involvement through self-selection effects by forensic experts in capital cases.

The present findings about evaluator self-selection factors due to death penalty beliefs raise the specter of skewed evaluator involvement in capital case evaluations. It appears that evaluators who are willing to do capital casework may support the death penalty more strongly than evaluators unwilling to do capital work or evaluations for the defense or court. Previous evidence demonstrated that examiner death penalty attitudes systematically influenced perceptions of hypothetical death row inmates [14, 27–28]. These data, in combination with mounting evidence of bias in forensic evaluations (see e.g., [9], [29–30]), highlight two research needs: to examine how evaluator attitudes and beliefs systematically influence reasoning processes and to develop methods to reduce the effects of systematic bias.

Stronger death penalty support was clearly associated with examiners being more willing to be involved in capital cases, evidenced both by the t-tests directly testing the hypothesis and in the multinomial logistic regression results (see the sharp and positively-curving red line at the top of the graph in Fig 1). As previously mentioned, each one-unit increase in death penalty support more than tripled the odds that an examiner would accept any capital referral compared to accepting just from the defense or defense-and-court. These findings—that psychologists who oppose the death penalty may be disproportionately absent from death penalty evaluations–are consistent with Haney’s [16] observation about the death-qualification of juries in capital cases. In both, capital proceedings are likely to include opinion formers and decision makers (whether forensic expert or juror) who are disproportionately accepting of the death penalty, compared to their broader populations. Another way of interpreting the results is more optimistic. Although statistically significant and meaningful selection bias is evident in the results as discussed above, an important piece of the story is that forensic psychologists as a group appear to be largely open-minded about being involved adversarially–even in capital cases. By far most of the sample fell into just one of the five outcome groups– 68% were in the “accept from any source” group. Clearly then, most forensic psychologists report they are willing to be involved in forensic cases in a non-adversarially-biased manner.

It should be noted that this paper takes a narrow methodological and analytical approach to answering a broad question about expert bias in the legal system. However, the broader question of how experts negotiate their moral perspectives and motivations in the legal framework in which their expert evidence must be given is much more complicated and nuanced question than this strictly quantitative and narrow approach can answer. Both broader qualitative and narrow quantitative approaches are valuable for answering questions about expert bias, and there are pros and cons to taking a narrow statistical approach like the current paper versus a broader, more textured, and qualitative approach to the same kind of questions. There are other recent papers in this line of research that take the broader, more textured, and qualitative approach to exploring expert bias in the legal system for any readers interested in a fuller picture.

For example, Neal and Grisso [30] wrote a paper wrestling with the definition of “bias” in forensic experts and what those kinds of biases might look like in the forensic mental health context. As another example–and one with data–Neal and Brodsky [31] integrated qualitative and quantitative methods across two studies to explore the relation between the occupational socialization process in forensic psychology and psychologists’ beliefs about their objectivity, finding that higher occupational socialization is associated with a stronger belief in one’s ability to be objective. And Neal and Brodsky [29] conducted both a qualitative and quantitative study of forensic psychologists’ perceptions of bias and potential correction strategies in forensic mental health evaluations. They found evidence of a “bias blind spot” in forensic evaluators, which is the tendency for experts to perceive themselves as less vulnerable to bias than their colleagues [32]; identified recurring situations that forensic psychologists reported pose challenges for their objectivity; and generated a list of 25 debiasing strategies that emerged from the qualitative study. They categorized those 25 strategies into three groups: literature-identified effective strategies perceived as useful by forensic clinicians, literature-identified ineffective strategies nevertheless perceived as useful by forensic clinicians, and new debiasing strategies identified by forensic clinicians that have not yet been subject to empirical investigation. They also outlined literature-identified effective strategies not mentioned by forensic clinicians.

In addition to the issue of qualitative vs. quantitative methods, one of the reviewers was concerned that this analysis comes from a narrow disciplinary perspective, pointing out that the questions with which this manuscript is concerned might also be studied from other disciplinary perspectives, such as the sociology of professions and the legal literature on death penalty jurisprudence. The larger project from which this data is drawn included a three-part project in which I approached the question “are forensic psychologists biased in capital cases” from an interdisciplinary and multi-method approach [33]. The sociology of occupational socialization and its relevance for understanding how forensic psychologists come to highly value and believe in their objectivity was the focus of Neal and Brodsky [31]. And Neal and Brodsky [29] discussed at length and in great detail forensic psychologists’ feelings, motivations, and understandings of bias in capital cases.

With regard to limitations in the current paper, the respondents were volunteers interested enough in the request for participation that they completed the survey. Although we identified a large sample of forensic clinicians and obtained a high response rate, participants were identified through the APA directory and thus everyone in this sample was a member of the APA or one of its divisions. Because not all forensic clinicians are members, the sample may not fully represent the population of forensic clinicians. Future studies should include forensic mental health professionals who are not members of professional associations. In addition, we asked forensic evaluators to rate their death penalty attitudes in the same packet of questionnaires in which we asked about willingness to be involved in capital case work. Therefore, it is possible that some responses were affected by socially desirable responding. Another limitation is that this survey asked clinicians about their willingness to accept various capital case referrals rather than studying the real-world behavior of clinicians in accepting or declining capital case referrals. Future studies should investigate actual involvement with these cases. Finally, the survey did not ask whether evaluators had ever declined a referral because of the party that requested the evaluation. Those data would usefully supplement these findings.

Future research is needed to explore how pre-selection affects experts’ information processing and whether it transfers to biased decision-making. Strong convictions for or against capital punishment may influence the examiner’s reasoning process, potentially skewing pro- death penalty experts’ opinions toward death outcomes and anti- death penalty experts’ opinions toward life outcomes. Other evaluator attitudes and attributes relevant to the issues in a given case may affect reasoning processes and conclusions in other ways as well. Some might argue that even if pre-existing bias affects choice of adversarial party for whom to work that scientific or professional objectivity would limit the effect of bias on the experts’ decision processes. However, a large and growing body of literature challenges this assumption (see e.g., [9], [30], [34–36]). In sum, gaining a better understanding of the limitations of expert witness objectivity may motivate the discovery of effective debiasing strategies for experts to use, as well as motivate the legal system to change the way it uses expert witnesses or the assumptions it makes about expert objectivity, all toward the ultimate goal of improving psycholegal opinions and just outcomes in legal cases.

## Supporting Information

S1 File Forensic Psychologist Questionnaire. (PDF)
