# Supplementary material for: Are Forensic Experts Already Biased before Adversarial Legal Parties Hire Them?
Source: PLoS One. 2016 Apr 28;11(4):e0154434. doi: 10.1371/journal.pone.0154434 (PMC4849669; doi:10.1371/journal.pone.0154434)
Supplement: S1 File — (PDF) [file pone.0154434.s001.pdf]

Neal, T.M.S. (2016). Are forensic experts already biased before adversarial legal parties hire them? *PLOS ONE.*
Supplemental document.

S1 File. Forensic Psychologist Questionnaire

1. What is the highest degree you have earned?
   __ Ph.D. __ J.D. __ Joint Ph.D./J.D.
   __ Psy.D. __ Master's Degree __Other (Specify______________)

2. What is your primary place of employment?
   a. __ Institution or agency (e.g., hospital, prison, court clinic, etc.)
      i. Specify type of institution____
   b. __ Private
      i. Specify type of practice____
   c. __ University
   d. __ Other or more than one
      (specify____)

3. Do you conduct forensic evaluations? __ Yes __ No

4. If so, for how many years have you conducted forensic evaluations? _____

5. Are you certified by a specialty board in forensic or clinical psychology? __ Yes __ No
   a. If yes, specify____

6. What is the primary state in which you practice? _________________________________

7. Are you licensed to practice in other states? __ Yes __No
   a. If so, in what other states are you licensed to practice?
      ____
      ____

8. Do any of the states in which you practice have the death penalty? __ Yes __No
   a. If so, which state(s)?____

9. If you practice in a death penalty state, do you perform capital case evaluations? _Yes _No

10. If a state in which you practice has the death penalty, have you or would you evaluate a defendant in a capital case for the prosecution? __ Yes __No

11. If the state in which you practice has the death penalty, have you or would you evaluate a defendant in a capital case for the defense? __ Yes __No

12. If applicable, have you or would you evaluate a defendant in a capital case for the court as a court appointed assessor? __ Yes __No

13. What is your gender? __Male __Female

14. What is your age? ______

15. What do you consider to be your race or ethnicity?

__ African American	__ Hispanic (non-white)	__ Pacific Islander
__ Asian	__ Hispanic (white)	__ White
__ Biracial	__ Native American	__ Other (Specify______________)
